# A 27 kDa heat shock protein that has anomalous prognostic powers in early and advanced breast cancer.

**DOI:** 10.1038/bjc.1994.140

**Published:** 1994-04

**Authors:** S. Love, R. J. King

**Affiliations:** Imperial Cancer Research Fund, Medical Statistics Laboratory, London, UK.

## Abstract

This paper describes a prospective immunohistochemical analysis of 27 kDa heat shock protein (HSP27) in 361 patients with primary breast cancer in relation to disease-free survival (DFS) and survival from first relapse (SR). Oestradiol (ER) and progesterone (PR) receptors were also quantitated and related to the HSP27 data. While ER positively predicted a good outcome for both DFS and SR, HSP27 positivity predicted a prolonged SR but short DFS. The association between HSP27 and DFS only attained statistical significance in node-negative patients. Subgroup analysis reinforced the complementary relationship of HSP27 and ER for SR and opposing influences for DFS. In both node-negative and node-positive women, ER+ HSP27- patients had a longer DFS than ER- HSP27+ counterparts. There was no relationship between HSP27 and overall survival. HSP27 staining was highly correlated with ER but not PR, patient age, tumour size or menstrual status. There was a marginal correlation (P = 0.04) with histological grade with well-differentiated tumours having the highest HSP27. Cox multivariate regression analysis of the contribution of HSP27 in the presence of data on ER, PR, stage, nodal status and histological grade indicated that HSP27 was not of independent prognostic importance for DFS or overall survival and was only of borderline significance for OS (P < 0.07). However, in the absence of ER and PR data, HSP27 staining is an effective way of getting the same prognostic information. HSP27 staining appears to correlate with different biological features in early and advanced breast, high HSP27 being linked with short DFS in node-negative patients but with prolonged survival from first recurrence.


Br. J. Cancer (1994), 69, 743-748 © Macmillan Press Ltd., 1994

### A 27 kDa heat shock protein that has anomalous prognostic powers in early and advanced breast cancer

S. Love[1] & R.J.B. King[2]

[1]*Imperial Cancer Research Fund, Medical Statistics Laboratory, 61 Lincoln's Inn Fields, London WC2A 3PX, UK;* [2]*Imperial Cancer Research Fund – Breast Biology Group, School of Biological Sciences, University of Surrey, Guildford, Surrey GU2 5XH, UK.*

**Summary** This paper describes a prospective immunohistochemical analysis of 27 kDa heat shock protein (HSP27) in 361 patients with primary breast cancer in relation to disease-free survival (DFS) and survival from first relapse (SR). Oestradiol (ER) and progesterone (PR) receptors were also quantitated and related to the HSP27 data. While ER positively predicted a good outcome for both DFS and SR, HSP27 positivity predicted a prolonged SR but short DFS. The association between HSP27 and DFS only attained statistical significance in node-negative patients. Subgroup analysis reinforced the complementary relationship of HSP27 and ER for SR and opposing influences for DFS. In both node-negative and node-positive women, $ER^+$ $HSP27^-$ patients had a longer DFS than $ER^-$ $HSP27^+$ counterparts. There was no relationship between HSP27 and overall survival. HSP27 staining was highly correlated with ER but not PR, patient age, tumour size or menstrual status. There was a marginal correlation ($P = 0.04$) with histological grade with well-differentiated tumours having the highest HSP27. Cox multivariate regression analysis of the contribution of HSP27 in the presence of data on ER, PR, stage, nodal status and histological grade indicated that HSP27 was not of independent prognostic importance for DFS or overall survival and was only of borderline significance for OS ($P < 0.07$). However, in the absence of ER and PR data, HSP27 staining is an effective way of getting the same prognostic information. HSP27 staining appears to correlate with different biological features in early and advanced breast, high HSP27 being linked with short DFS in node-negative patients but with prolonged survival from first recurrence.

Much effort has been directed at analysing hormone and growth factor receptors, oncogene products and indices of cell proliferation in human breast cancers with results of both biological and clinical importance (Sutherland & McGuire, 1991; Davidson & Abeloff, 1992). We have been interested in a different type of protein, a low molecular weight (27 kDa) heat shock protein (HSP27). This is an abundant protein that has previously been discussed under three different names, p29 (King, 1986; King *et al.*, 1987), p24 (Ciocca *et al.*, 1983; Adams & McGuire, 1985) and srp27 (Thor *et al.*, 1991), but it is now known that they are one and the same protein (Mendelsohn *et al.*, 1991; Ciocca & Luque, 1991) and will henceforth be called HSP27. HSP27 is a widely distributed protein, although levels attained in breast cancers exceed those found in other cells (Dunn *et al.*, 1993). Its functions are unknown although by analogy with the larger members of the heat shock protein family it may be involved in regulating the structure, intracellular transport or secretion of other proteins (Georgopoulos, 1992; Gething & Sambrook, 1992). A number of reports link HSP27 expression with certain types of drug resistance (Dunn *et al.*, 1993), viral neoplasia (Zantema *et al.*, 1989) and a range of normal cell functions (Dunn *et al.*, 1993), but it is difficult to get a clear picture of the biological roles of HSP27 from these fragmentary data. Interest in this protein has derived from several separate lines of work. Its association to oestrogen action has been most extensively studied as HSP27 is qualitatively and quantitatively related to ER in a range of oestrogen-sensitive cells including primary breast cancers (Dunn *et al.*, 1993). Like ER, HSP27 predicts for hormone sensitivity of advanced breast cancers (King *et al.*, 1987) and will react with specific forms of ER (Coffer *et al.*, 1985*a*; Coffer & King, 1988). Furthermore, HSP27 is expressed in increased amounts in invasive breast cancers as compared with carcinoma *in situ*, which in turn has more than normal mammary epithelium (King *et al.*, 1987; Girling *et al.*, 1988).

It has also been reported that elevated levels of HSP27 are prognostic for rapid recurrence of breast cancer (Tandon *et al.*, 1990; Thor *et al.*, 1991), which is at variance with its positive correlation with ER, high levels of which predict for long disease-free survival (McGuire, 1987; Jordan *et al.*, 1988). No previous publication comments on this anomaly or presents data on survival from first recurrence. From the correlation with ER and with response to hormones, high levels of HSP27 would be expected to indicate long such survival.

To clarify the role of HSP27, we studied the effects of HSP27 and ER on disease-free survival, survival from first recurrence and overall survival on a prospective series of 361 primary breast cancers from women attending a single clinic.

### Materials and methods

### *Patients*

Tumours were obtained over a 3 year period from patients with operable breast cancer treated either by modified radical mastectomy or by excision of tumour followed by radiotherapy. The only tumours omitted were those for which histopathology and ER analysis utilised all the material. Of the 452 tumours assembled, 91 were excluded for having locally advanced or metastatic disease (86), carcinoma *in situ* (4) or loss to follow-up (1). This analysis is therefore based on 361 patients with stage 1 or 2 disease. Median follow-up from diagnosis (using the time when the Kaplan–Meier plot, with event status reversed, passes through 50%) was 6 years 2 months.

### *Tissues and staining*

Tissues were fixed in methacarn (methanol–chloroform–acetic acid; 60:30:10) and immediately processed as described previously (Cano *et al.*, 1986). The present data are primarily derived from the samples described in that report. The monoclonal antibody D5 against HSP27 has been described elsewhere (Coffer *et al.*, 1985; King & Coffer, 1986). It is human specific and in immunoblots of tissue extracts recognises only HSP27. In histochemistry it recognises HSP27 in alcohol- and methacarn-fixed tissues, but is ineffective with other fixatives. Sections were stained for 1 h with antibody

Correspondence: R.J.B. King.
Received 12 July 1993; and in revised form 8 November 1993.

D5 (8 μg of protein per ml) and, after washing for 45 min, with a 1:50 dilution of peroxidase-conjugated sheep anti-mouse IgG (Amersham International, Amersham, UK) containing 1:25 dilution of human serum. Peroxidase was identified with diaminobenzidine plus hydrogen peroxide.

### *Quantitation of staining in breast tumour sections*

Tumour cellularity, staining intensity and proportion of positive tumour cells were assessed by eye as previously described (Cano *et al.*, 1986). The proportion of tumour cells per section was assessed by eye and allocated a score of 0–6. Staining intensity was estimated on a 0–3 + scale. The staining index was a simple multiple of the cellularity and staining scores. This method of quantitation was selected to successfully facilitate comparison with biochemical assays, which are a combination of antigen per cell and no. of antigen-containing cells (Cano *et al.*, 1986). As described in the Results section, some statistical comparisons were made in which either stain intensity or cellularity was assessed independently. In no case did these methods of quantitation alter the conclusions obtained by using stain index. Two sections were stained, and values calculated as a mean of the two individual values. Unless stated otherwise a staining index of greater than 2.0 was taken as positive. This value was selected on the basis of being the lowest value at which separate observers agreed a sample was positive. The index value of 7 used to separate high and moderate HSP27 staining was selected as the value above which the highest percentage of tumours responded to hormone therapy (data not shown).

### *Oestradiol and progesterone receptor assays*

Ligand-binding assays were performed as previously described (King *et al.*, 1977). A value of ⩾20 fmol per mg of protein was taken as positive.

### *Statistical methods*

For disease-free survival (DFS), time was measured from histological diagnosis to recurrence, death or date of last follow-up. Patients who died from a known cause other than breast cancer, with the patients thought to be in remission, and those not known to have relapsed or died, had censored survival times. For survival from first recurrence (SR), time was measured from date of recurrence to death or date of last follow-up. Events were death from breast cancer, death with breast cancer present and uncertain cause of death. Patients dying from a known cause, with their breast cancer thought to be in remission, and those not known to have died, had censored survival times.

For overall survival (OS), time was measured from date of diagnosis to death or last follow-up. Events were death from breast cancer, death with breast cancer present and uncertain cause of death. Patients dying from a known cause with breast cancer in remission and those not known to have died had censored survival times.

Graphs of survival were drawn using the Kaplan–Meier method and univariate survival analysis of categorical variables was by the log-rank test (Peto *et al.*, 1977). For continuous variables, univariate Cox analysis was used to avoid setting cut-off points (Cox, 1972). Variables significant at $P = <0.1$ in the univariate analysis were put into a stepwise Cox regression model to discover which were the strongest independent predictors. In the Cox model, a negative coefficient indicates a positive relationship of the variable with survival and vice versa.

Progesterone receptors had a skew distribution, so to avoid the few large values having an unduly large effect the natural logarithm of progesterone receptor is used in the Cox regression analysis.

### Results

### General characteristics

HSP27 staining correlated with ER but not PR, age, tumour size, nodal status, stage or menstrual status (Table I). Histological grade 1 tumours had lower HSP27 staining than the less well-differentiated grade 2 and 3 tumours. Twenty-seven per cent of all tumours had low HSP27, while the proportions of the four ER, HSP27 phenotypes were $ER^+$ $HSP27^+$ 66%, $ER^-$ $HSP27^-$ 9%, $ER^+$ $HSP27^-$ 18% and $ER^-$ $HSP27^+$ 7%.

**Table I** Correlation of HSP27 with other variables

| | *Correlation coefficient* | *P-value* |
|---|---|---|
| Continuous variable | | |
| ER | 0.245 | 0.001 |
| PR | 0.105 | >0.05 |
| Age | – 0.06 | >0.05 |
| Tumour size | – 0.08 | >0.05 |
| Discrete variable[a] | | |
| Stage | 5.87 | 0.05 |
| Nodal status | 1.43 | 0.2 |
| Menstrual status | 0.77 | 0.9 |
| Histological grade | 6.36 | 0.04 |

[a]Kruskal–Wallis test.

**Figure 1** Influence of oestradiol receptor **a** and HSP27 **b** levels on disease-free survival. **a**, Values are fmol per mg of protein. The numbers of patients at risk at 5 years are 0–9 = 25, 10–19 = 9, 20–999 = 119. Chi-square 8.005, $P = 0.018$. **b**, Values are stain index as defined in Materials and methods. The numbers of patients at risk at 5 years are 0–2 = 46, 2.1–7 = 73, 7.1–25 = 42. Chi-square 3.49, $P = 0.175$.

*Disease-free survival (DFS)*

There were 361 patients and 174 events. Considering all patients, ER (Figure 1a) was significantly associated with DFS (univariate Cox, coefficient = − 0.001, standard error 0.0004, $P = 0.002$) and HSP27 (Figure 1b) was approaching significance (univariate Cox, coefficient = 0.03, standard error = 0.017, $P = 0.07$). However, the coefficient for ER is negative, indicating a positive effect with DFS, and the coefficient for HSP27 is positive, indicating a negative effect with survival. This was unexpected given the correlation between ER and HSP27 and between high ER and long DFS. HSP27 was only prognostic in node-negative patients (Figure 2).

As high ER and HSP27 had opposing influences on DFS in the node-negative group, their combined effects were analysed in all patients and the separate nodal subgroups. The $ER^+$ $HSP27^-$ phenotype exhibited the longest DFS, whereas the $ER^-$ $HSP27^+$ tumours recurred more rapidly. This was true for all patients (Figure 3a) and after separation into node-negative (Figure 3b) and -positive (Figure 3c) subgroups. In the node-negative group the worst prognosis was exhibited by $ER^-$ $HSP27^+$ patients, whereas $ER^-$ $HSP27^-$ patients had the worst prognosis in the node-positive group. Patients with $ER^+$ $HSP27^+$ tumours did worse than those with $ER^-$ $HSP27^-$ tumours in all three categories. In the node-positive patients, ER phenotype exerted a stronger influence on tumour behaviour than HSP27, as both the $ER^+$ $HSP27^+$ and $ER^+$ $HSP27^-$ categories had longer DFS than the $ER^-$ $HSP27^+$ and $ER^-$ $HSP27^-$ groups. However, using the best method of considering differing influences of ER and HSP27 in nodal subgroups (i.e. by including an interaction term in a Cox model), it would not be concluded that the effects in the nodal subgroups differed (i.e. the interaction term was not significant).

*Survival from first recurrence (SR)*

There were 171 patients and 114 events. Both ER (Figure 4a) and HSP27 (Figure 4b) were significantly correlated with SR (univariate Cox, coefficient for ER = − 0.002, standard

**Figure 2** Influence of HSP27 levels on disease-free survival in node-positive **a** or node-negative **b** patients. HSP levels are as defined in Figure 1. The numbers of patients at risk at 5 years are **a**, 0–2 = 9, 2.1–7 = 21, 7.1–25 = 16. Chi-square 0.203, $P = 0.903$. **b**, 0–2 = 30, 2.1–7 = 40, 7.1–25 = 20. Chi-square 7.293, $P = 0.026$.

**Figure 3** Influence of combined oestradiol receptor (ER) and HSP27 (I) phenotypes on disease-free survival in: **a**, all patients; **b**, node-negative patients; **c**, node-positive patients. ER + > 20 fmol $mg^{-1}$ protein; I = > 2 index. The numbers of patients at risk at 5 years are: **a**, $ER^+$ $I^+$ = 105, $ER^+$ $I^-$ = 31, $ER^-$ $I^-$ = 15, Chi-square 8.119, $P = 0.044$; **b**, $ER^+$ $I^+$ = 53, $ER^-$ $I^+$ = 7, $ER^+$ $I^-$ = 18, $ER^-$ $I^-$ = 12, Chi-square 8.682, $P = 0.034$; **c**, $ER^+$ $I^+$ = 35, $ER^-$ $I^+$ = 2, $ER^+$ $I^-$ = 8, $ER^-$ $I^-$ = 1, Chi-square 16.68, $P = < 0.001$.

error = 0.0006, $P = 0.003$, coefficient for HSP27 = − 0.081, standard error = 0.0245, $P = 0.001$). In contrast to DFS, both coefficients are negative, indicating that both ER and HSP27 have a positive effect with SR. The distinction between SR and DFS was accentuated when the combined ER and HSP27 phenotypes were compared (Figure 5). HSP27 behaved as a surrogate for ER such that either ER or HSP27 positivity indicated a longer SR than a negative phenotype in the long (>4 years) but not short term (<3 years).

**Figure 4** Influence of **a** oestradiol receptor and **b** HSP727 levels on survival from first relapse. Abbreviations are as described in Figure 1. The numbers of patients at risk at 5 years are: **a**, 0–9 = 1, 10–19 = 1, 20–999 = 26, Chi-square 14.64, $P = <0.001$. **b**, 0–2 = 4, 2.1–7 = 8, 7.1–25 = 16, Chi-square 12.71, $P = 0.002$.

**Table II** Overall survival

| *Variable* | *No. of patients* | *Log rank* | *P-value* |
|---|---|---|---|
| HSP27 | 360 | 0.9 | 0.7 |
| ER | 346 | 15.3 | <0.001 |
| PR | 331 | 15.3 | <0.001 |
| Stage | 361 | 35.8 | <0.001 |
| Nodal status | 361 | 32.3 | <0.001 |
| Age | 361 | 0.2 | 0.9 |
| Tumour size | 349 | 13.6 | 0.003 |
| Grade | 282 | 12.2 | 0.01 |

HSP27 stain index is a multiple of cellularity and stain intensity. Where stain index was significant, staining intensity and cellularity were separately assessed. For the SR univariate analysis, stain intensity was significant (coefficient = − 0.293, standard error = 0.0958, $P = 0.003$) but cellularity only approached significance (coefficient = 0.203, standard error = 0.1110, $P = 0.07$). Considering the multivariate analysis (see below) for SR, staining intensity could replace the index with little difference in the fit of the model or the other covariates (data not shown).

### *Overall survival*

There were 361 patients and 117 events. As high HPS27 predicts for short DFS but long SR, HSP27 has no relationship to overall survival (univariate Cox, coefficient = − 0.016, standard error 0.0224, $P = 0.5$), whereas highly significant effects of ER, PR, stage, nodal status (all $P < 0.001$) and histological grade ($P = 0.01$) were obtained (Table II).

### *Multivariate analysis*

Putting all the variables with $P < 0.1$ in univariate analysis into a stepwise Cox proportional hazards regression procedure gives a final model containing the variables showing an independent prognostic importance. HSP27 is not of indpendent prognostic importance for DFS or OS (forcing this covariate into either model gives $P > 0.2$) and is only of

**Figure 5** Influence of combined oestradiol receptor (ER) and HSP27 (I) phenotypes on survival from first relapse. Abbreviations are as described in Figure 3. The numbers of patients at risk at 5 years are: $ER^+$ $I^+$ = 23, $ER^-$ $I^+$ = 1; $ER^+$ $I^-$ = 4, $ER^-$ $I^-$ = 0, Chi-square 20.92, $P < 0.01$.

**Table III** Coefficient of HSP27 (s.e.) in multivariate Cox regression model

| *Variables* | *Disease-free survival* | *Survival from first relapse* | *Overall survival* |
|---|---|---|---|
| All[a] | NS | − 0.051 (0.029)[b] $P = 0.07$ | NS |
| Omit grade | NS | NS | NS |
| Omit ER and PR | NS | − 0.105 (0.027) $P = 0.0001$ | − 0.053 (0.025) $P = 0.03$ |
| Omit grade, ER and PR | NS | − 0.109 (0.027) $P < 0.0001$ | − 0.052 (0.025) $P = 0.04$ |

[a]Grade, ER, PR, nodal status, menstrual status, age, tumour size and histology. [b]A negative coefficient indicates a positive effect with survival. Standard errors are contained within the brackets. NS, not significant; s.e. standard error.

borderline significance in SR (coefficient = − 0.0512, standard error = 0.0287, $P = 0.07$). In this latter case, the coefficient being negative means that a higher value for HSP27 increases survival.

Multivariate analysis was also used to see if HSP27 was of independent prognostic importance in four particular circumstances (Table III): (i) with grade, ER and PR available; (ii) with ER and PR available but not grade; (iii) with grade available, no ER or PR; (iv) with none of grade, ER or PR available.

In all cases index, nodal status, menstrual status, age, tumour size, and histology were available.

From this table, adjusting for other variables as indicated, index is independently prognostic for SFR or OS, with low HSP27 indicating poor survival only when ER and PR are not available. Even if neither grade, ER or PR is available, HSP27 is not independently prognostic for DFS.

### Discussion

There are clearly divergent influences of HSP27 on DFS and SR, the reasons for which are unknown. The positive association between high HSP27 staining and long SR agrees well with the published literature on its association with ER (Dunn *et al.*, 1993) and their ability to predict for hormone responsiveness of advanced breast cancer (King *et al.*, 1987). Women with breast cancers that respond to hormone treatment have a longer survival from first relapse than those with unresponsive tumours. Also, the correlation of high HSP27 with short DFS reported here is in broad agreement with the previous publication of Thor *et al.* (1991), although differences exist in respect of the nodal groups. For HSP27 alone we found an effect in node-negative but not -positive patients, whereas Thor *et al.* (1991) obtained the opposite result and Tandon *et al.* (1991) reported the same result as ourselves. Danestrup *et al.* (1991) found no correlation between HSP27 and disease-free survival.

None of these publications commented on the biological behaviour of different HSP27 and ER phenotypes. Combination of these two variables generated significant differences in DFS among the four phenotypes in both node-negative and -positive groups. As $ER^+$ $HSP27^+$ tumours recur more rapidly than the $ER^+$ $HSP27^-$ ones, a positive link between HSP27 and growth rate is plausible, which is supported by other data. Antisense oligonucleotides directed against HSP27 mRNA inhibit the proliferation of $ER^+$, ZR75 human breast cancer cells in parallel with decreased HSP27 levels (D.K. Dunn & R.J.B. King, in preparation), while high levels of HSP27 in normal endometrial epithelium (King *et al.*, 1987) and normal mammary epithelium (King *et al.*, 1993) correlate with proliferative activity at different stages of the menstrual cycle. Those data on normal epithelia would also suggest that HSP27 is linked to proliferation rather than to a specific hormonal environment as endometrial proliferation is stimulated by oestrogen whereas breast correlates with a progestational state (King, 1992). Interestingly, high HSP27 also correlates with rapid recurrence of gastric cancer (Harrison *et al.*, 1991) so its relevance may extend beyond endocrine-related cancers. These data linking high HSP27 with rapid proliferation could provide an explanation for the former's correlation with short DFS, but why this should be confined to node-negative patients remains an enigma. Possibly other biological features associated with metastasis override the proliferation effect. However, the putative link between HSP27 and proliferation cannot explain the positive correlation between high HSP27 hormone sensitivity and good survival from first recurrence; in general, hormone-sensitive breast tumours proliferate more slowly than their insensitive counterparts (McGuire, 1987). This link with hormone response is nevertheless compatible with the association between HSP27 and ER discussed earlier.

The switch from HSP27 being a bad to a good prognostic factor in early and advanced breast cancer respectively must have an as yet unidentified biological explanation. Little is known about the function of HSP27, although its initial description as a heat shock protein links it to larger sized members of this class of proteins which have multiple functions related to many aspects of protein organisation and transport (Georgopoulos, 1992; Gething & Sambrook, 1992); beyond this it is not possible to formulate a unitary model for the role of HSP27 in cell function. It is increased by oestrogens in endometrial epithelium (King *et al.*, 1987) and some, but not all, breast cancer cell lines (Dunn *et al.*, 1993) and by progestins in endometrial stroma (Padwick *et al.*, 1988) and normal breast epithelium (King *et al.*, 1993), and is constitutively expressed in cervix (Hendry *et al.*, 1988). It is up-regulated in breast cancers (King *et al.*, 1987) and in virally transformed cells (Zantema *et al.*, 1989) and in some other cancers such as stomach (Harrison *et al.*, 1991) and brain cancer (Kato *et al.*, 1992) and certain leukaemias (Strahler *et al.*, 1991). HSP27 also has a complex relationship to chemotherapeutic drug sensitivity; drug resistance can be associated with increased (Huot *et al.*, 1987; Ciocca *et al.*, 1992) or decreased (Whelan & Hill, 1993; Dunn *et al.*, 1993) HSP27 depending on the drug in question and the experimental system used. The simplest explanation of these diverse correlations is that HSP27 is involved in many different cell functions, possible by influencing the structure of different proteins in different cells. This paper suggests that studies along these lines will be rewarding at both a basic and clinical level.

These data linking HSP27 with both growth and hormone sensitivity are of biological interest, but the ability to identify good and poor prognosis groups, especially in node-negative patients, may also have clinical relevance. In early-stage disease selection of high-risk patients, especially in node-negative women, is important in the context of not giving adjuvant treatment to women who have a high probability of long, normal life without it (McGuire, 1989; Sigurdsson *et al.*, 1990). Given the abundance of HSP27 and its simple, inexpensive immunohistochemical assay, its use as a prognostic factor for node-negative breast cancer should be considered. In advanced cancer high levels of HSP27 are indicative of a long survival probably because of the link with hormone response. ER assays have rightly been used to predict this feature (Jordan *et al.*, 1988) but our data reported here reinforce the previous suggestion (King *et al.*, 1987) that HSP27 may achieve the same objective and that, because of its abundance, lack of influence of age and possibly treatment regimens on amounts and ease of assay, HSP27 assays have some advantages over those for ER. If ER and PR values are not available, immunohistochemical evaluation of HSP27 is an effective method of obtaining equivalent clinical information.

We thank Rosemary Millis for supplying the tissue sections and pathology data and Walter Gregory (both Clinical Oncology Unit, Guy's Hospital) for abstracting the relevant clinical data from their database.

### References

ADAMS, D.J. & MCGUIRE, W.L. (1985). Quantitative enzyme-linked immunosorbent assay for the estrogen-regulated $M_r$ 24,000 protein in human breast tumors: correlation with estrogen and progesterone receptors. *Cancer Res.*, **45**, 2445–2449.

CANO, A., COFFER, A.I., ADATIA, R., MILLIS, R.R., RUBENS, R.D. & KING, R.J.B. (1986). Histochemical studies with an estrogen receptor-related protein in human breast tumors. *Cancer Res.*, **46**, 6475–6480.

CIOCCA, D.R. & LUQUE, E.H. (1991). Immunological evidence for the identity between the hsp27 estrogen-regulated heat shock protein and the p29 estrogen receptor-associated protein in breast and endometrial cancer. *Breast Cancer Res. Treat.*, **20**, 33-42.

CIOCCA, D.R., FUQUA, S.A.W., LOCK-LIM, S., TOFT, D.O., WELCH, W.J. & MCGUIRE, W.L. (1992). Response of human breast cancer cells to heat shock and chemotherapeutic drugs. *Cancer Res.*, **52**, 3648-3654.

CIOCCA, D.R., ADAMS, D.J., EDWARDS, D.P., BJERCKE, R.J. & MCGUIRE, W.L. (1983). Distribution of an estrogen-induced protein with a molecular weight of 24,000 in normal and malignant human tissues and cells. *Cancer Res.*, **43**, 1204-1210.

COFFER, A.I. & KING, R.J.B. (1988). Characterization of p29, an estrogen-receptor associated tumor marker. *J. Steroid Biochem.*, **31**, 745-750.

COFFER, A.I., LEWIS, K.M., BROCKAS, A.J. & KING, R.J.B. (1985). Monoclonal antibodies against a component related to soluble estrogen receptor. *Cancer Res.*, **45**, 3686-3693.

COX, D.R. (1972). Regression models and life tables. *J.R. Stat. Soc.* (B) **34**, 187-202.

DAMSTRUP, L., ANDERSEN, J., KUFE, D.W., HAYES, D.F. & SKOVGAARD POULSEN, H. (1992). Immunocytochemical determination of the estrogen-regulated proteins $M_r$ 24,000, $M_r$ 52,000 and DF3 breast cancer associated antigen: clinical value in advanced breast cancer and correlation with estrogen receptor. *Ann. Oncol.*, **3**, 71-77.

DAVISON, N.E. & ABELOFF, M.D. (1992). Adjuvant systemic therapy in women with early-stage breast cancer at high risk for relapse. *J. Natl Cancer Inst.*, **84**, 301-305.

DUNN, D.K., WHELAN, R.D.H., HILL, B. & KING, R.J.B. (1993). Relationship of hsp27 and oestrogen receptor in hormone sensitive and insensitive cell lines. *J. Steroid Biochem. Mol. Biol.*, **46**, 469-479.

GEORGOPOULOS, C. (1992). The emergence of the chaperone machines. *Trends Biol. Sci.*, **17**, 295-299.

GETHING, M.-J. & SAMBROOK, J. (1992). Protein folding in the cell. *Nature*, **355**, 33-45.

GIRLING, A., CALEFFI, M., KING, R.J.B. & MILLIS, R.R. (1988). Immunohistochemical study of D5 antigen (an oestrogen receptor related protein) in normal breast, benign breast disease, and mammary cardinoma *in situ*. *J. Clin. Pathol.*, **41**, 448-453.

HARRISON, J.D., JONES, J.A., ELLIS, I.O. & MORRIS, D.L. (1991). Estrogen receptor D5 antibody is an independent negative prognostic factor in gastric cancer. *Br. J. Surg.*, **78**, 334-336.

HENDRY, R.J.W., NICHOLAS, D.S., GOODMAN, J.D., GODLEY, M., RAJU, S., COFFER, A.I. & KING, R.J.B. (1988). Immunohistochemical study of cytoplasmic oestradiol receptor in normal, dysplastic and malignant cervical tissue. *Br. J. Obstet. Gynaecol.*, **95**, 927-932.

HUOT, J., ROY, G., LAMBERT, H., CHRÉTIEN, P. & LANDRY, J. (1991). Increased survival after treatments with anticancer agents of chinese hamster cells expressing the human $M_r$ 27,000 heat shock protein. *Cancer Res.*, **51**, 5245-5252.

JORDAN, V.C., WOLF, M.F., MIRECKI, D.M., WHITFORD, D.A. & WELSHONS, W.V. (1988). Hormone receptor assays: clinical usefulness in the management of carcinoma of the breast. *CRC Crit. Rev. Clin. Lab. Sci.*, **26**, 97-152.

KATO, K., HERZ, F., KATO, S. & HIRANO, A. (1992). Expression of stress-response (heat-shock) protein-27 in human brain-tumors – an immunohistochemical study. *Acta Neuropathol.*, **83**, 420-422.

KING, R.J.B. (1993). Estrogen and progestin effects in human breast carcinogenesis. *Breast Cancer Res. Treat.*, **27**, 3-15.

KING, R.J.B. & COFFER, A.I. (1986). The generation of antibodies against partially purified estradiol receptor from human myometrium. In *Estrogen/Antiestrogen Action and Breast Cancer Therapy*, Jordan, V.C. pp. 375-394. University of Wisconsin Press: Madison.

KING, R.J.B., HAYWARD, J.L., KUMAOKA, S. & YAMAMOTO, H. (1977). Comparison of soluble oestrogen and progestin receptor content of primary breast tumours from Japan and Britain. *Eur. J. Cancer*, **13**, 967-970.

KING, R.J.B., FINLEY, J.R., COFFER, A.E., MILLIS, R.R. & RUBENS, R.D. (1987). Characterization and biological relevance of a 29-kDa, oestrogen receptor-related protein. *J. Steroid Biochem.*, **27**, 471-475.

KING, R.J.B., BATTERSBY, S., ANDERSON, T.J. & MCPHERSON, K. (1993). Relationship of proliferation and HSP27 in normal human breast epithelium through the menstrual cycle. In preparation.

MCGUIRE, W.L. (1987). Prognostic factors for recurrence and survival in human breast cancer. *Breast Cancer Res. Treat.*, **10**, 5-9.

MCGUIRE, W.L. (1989). Adjuvant therapy of node-negative breast cancer. *N. Engl. J. Med.*, **320**, 525-527.

MENDELSOHN, M.E., ZHU, Y. & O'NEILL, S. (1991). The 29-kDa proteins phosphorylated in thrombin-activated human platelets are forms of the estrogen receptor-related 27-kDa heat shock protein. *Proc. Natl Acad. Sci. USA*, **88**, 11212-11216.

PADWICK, M.L., WHITEHEAD, M., COFFER, A. & KING, R.J.B. (1988). Demonstration of oestrogen receptor related protein in female tissues. In *The Menopause*, Studd, J.W.W. & Whitehead, M.I. (eds) pp. 227-233. Blackwell Scientific: Oxford.

PETO, R., PIKE, M.C., ARMITAGE, P., BRESLOW, N.E., COX, D.R., HOWARD, S.V., MANTEL, M., MCPHERSON, K., PETO, J. & SMITH, P.G. (1977). Design and analysis of randomised clinical trials requiring prolonged observation of each patient. II. Analysis and examples. *Br. J. Cancer*, **35**, 1-39.

SIGURDSSON, H., BALDETORP, B., BORG, Å. DALBERG, M., FERNÖ, M., KILLANDER, D. & OLSSON, M.D. (1990). Indicators of prognosis and node-negative breast cancer. *N. Engl. J. Med.*, **322**, 1045-1053.

STRAHLER, J.R., KUICK, R. & HANASH, S.M. (1991). Diminished phosphorylation of a heat shock protein (hsp27) in infant acute lymphoblastic leukemia. *Biochem. Biophys. Res. Commun.*, **175**, 134-142.

SUTHERLAND, M.C. & MCGUIRE, W.L. (1991). Oncogenes as clinical prognostic indicators: In: *Regulatory Mechanisms in Breast Cancer*. M.E. Lippman & R.B. Dickson (eds) pp. 3-22. Kluwer Academic: Boston.

TANDON, A.K., CLARK, G.M., CHAMNESS, G.C., FUQUA, S.A.W., WELCH, W.J., RIEHL, R.M. & MCGUIRE, W.L. (1991). Heat shock/stress response proteins: biological and clinical significance in breast cancer. *Proc. Am. Soc. Clin. Oncol.*, **9**, 84.

THOR, A., BENZ, C., MOORE, D., GOLDMAN, E., EDGERTON, S., LANDRY, J., SCHWARTZ, L., MAYALL, B., HICKEY, E. & WEBBER, L.A. (1991). Stress response protein (srp27) determination in primary human breast carcinomas: clinical, histologic and prognostic correlations. *J. Natl. Cancer Inst.*, **83**, 170-178.

WHELAN, R.D.H. & HILL, B.T. (1993). Differential expression of steroid receptors, HSP27 & pS2 in a series of drug resistant human breast cancer cell lines derived following exposure to antitumor drugs or to fractionated X-irradiation. *Breast Cancer Res. Treat.*, **26**, 23-93.

ZANTEMA, A., JONG, E. DE., LARDENOIJE, R. & VEN DER EV, A.J. (1989). The expression of heat shock protein hsp27 and a complexed 22-kilodalton protein is inversely correlated with the oncogenicity of adenovirus-transformed cells. *J. Virol.*, **63**, 3368-3375.


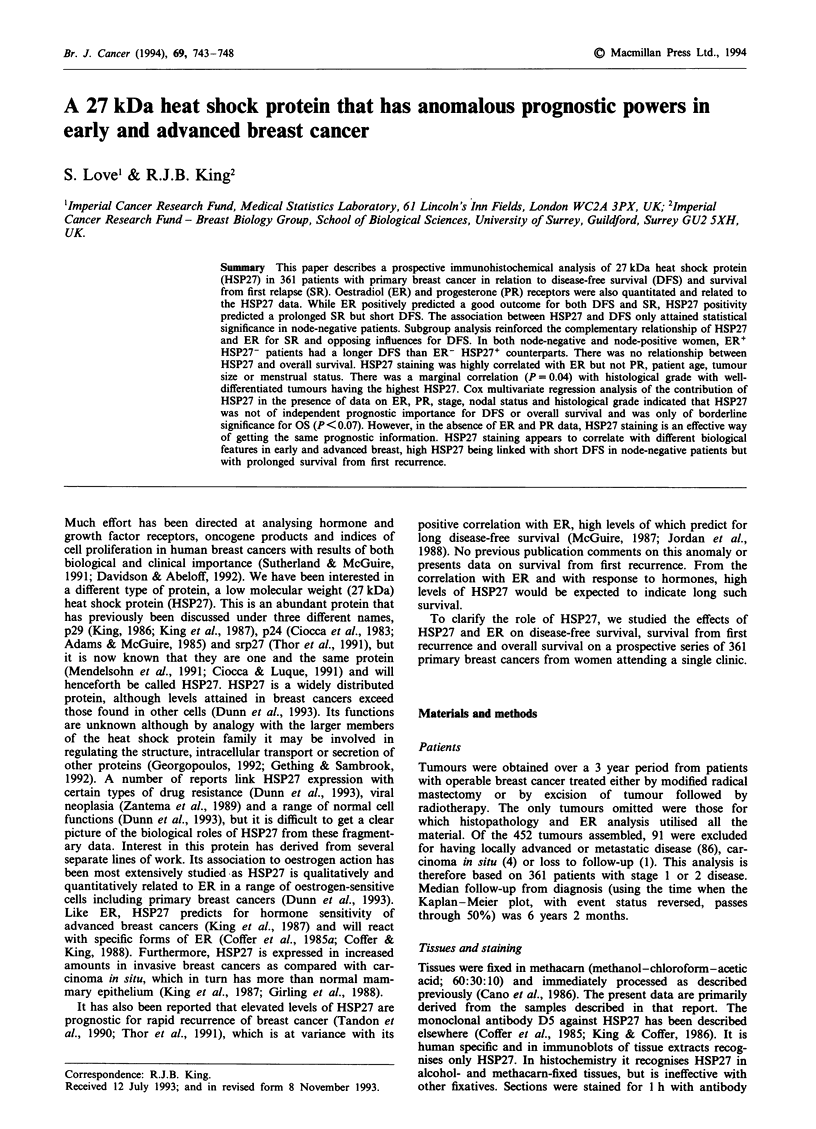

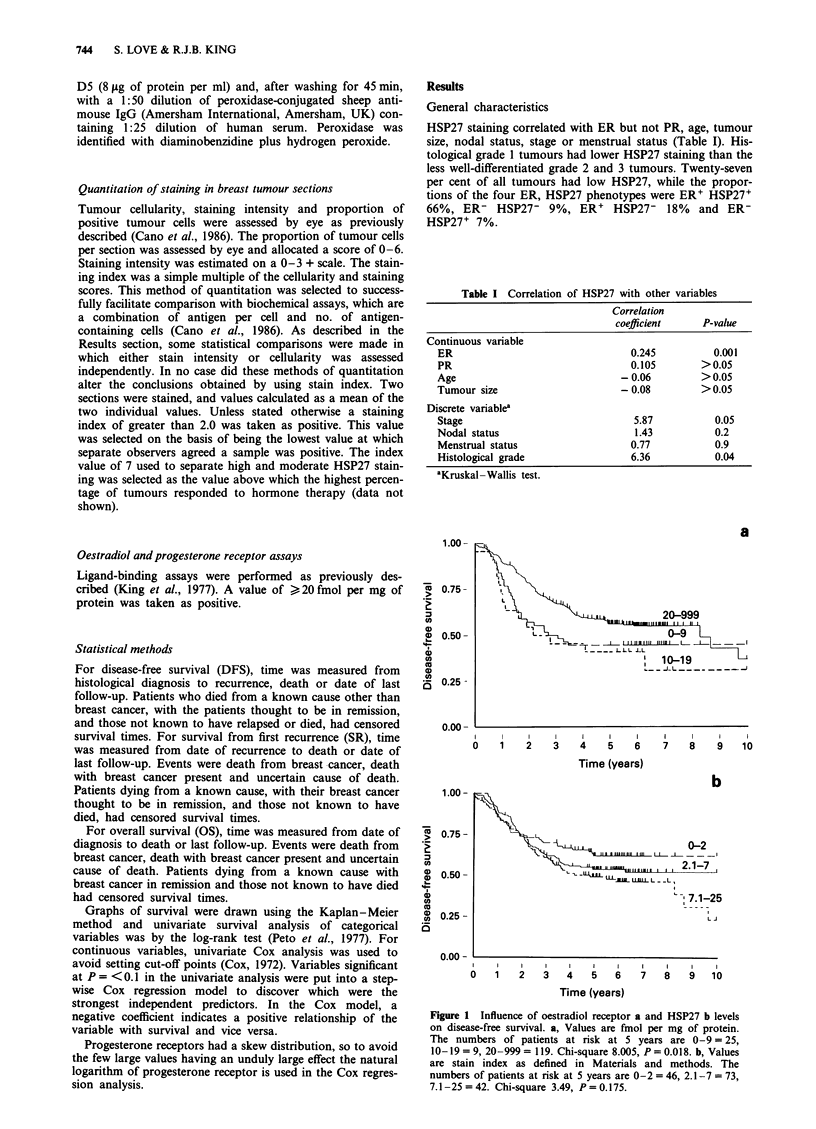

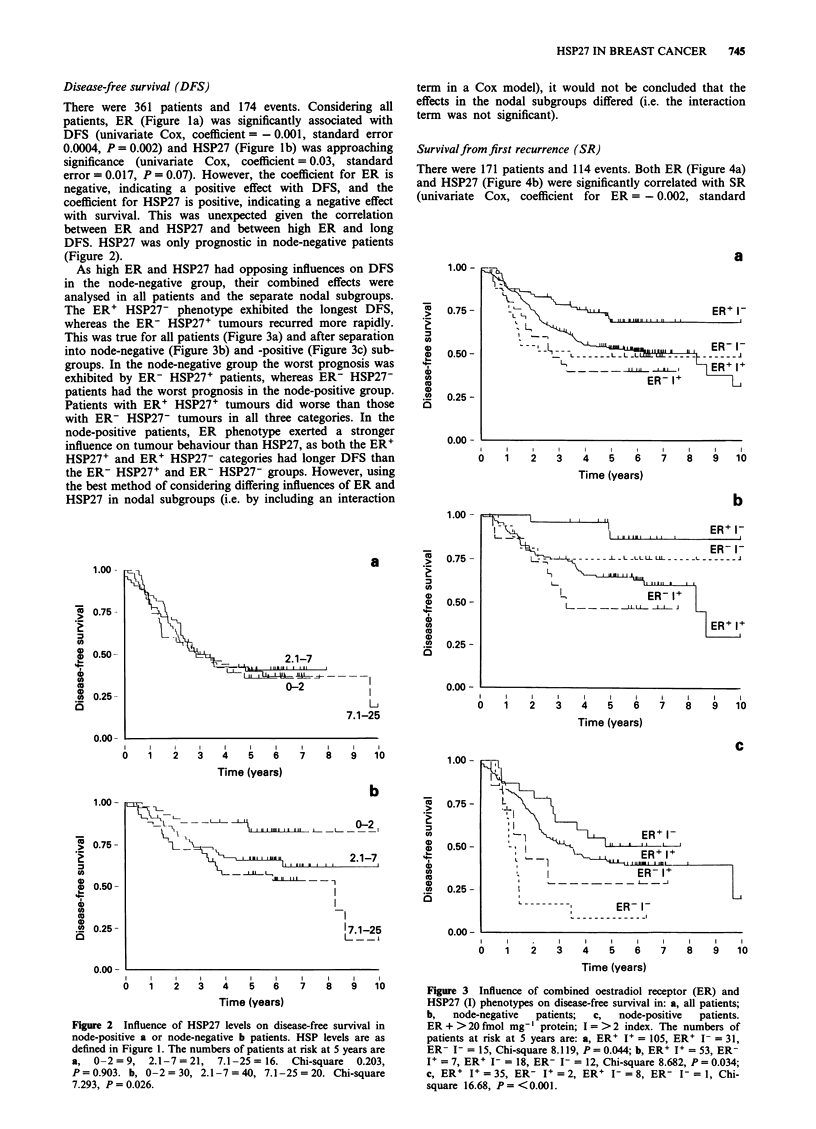

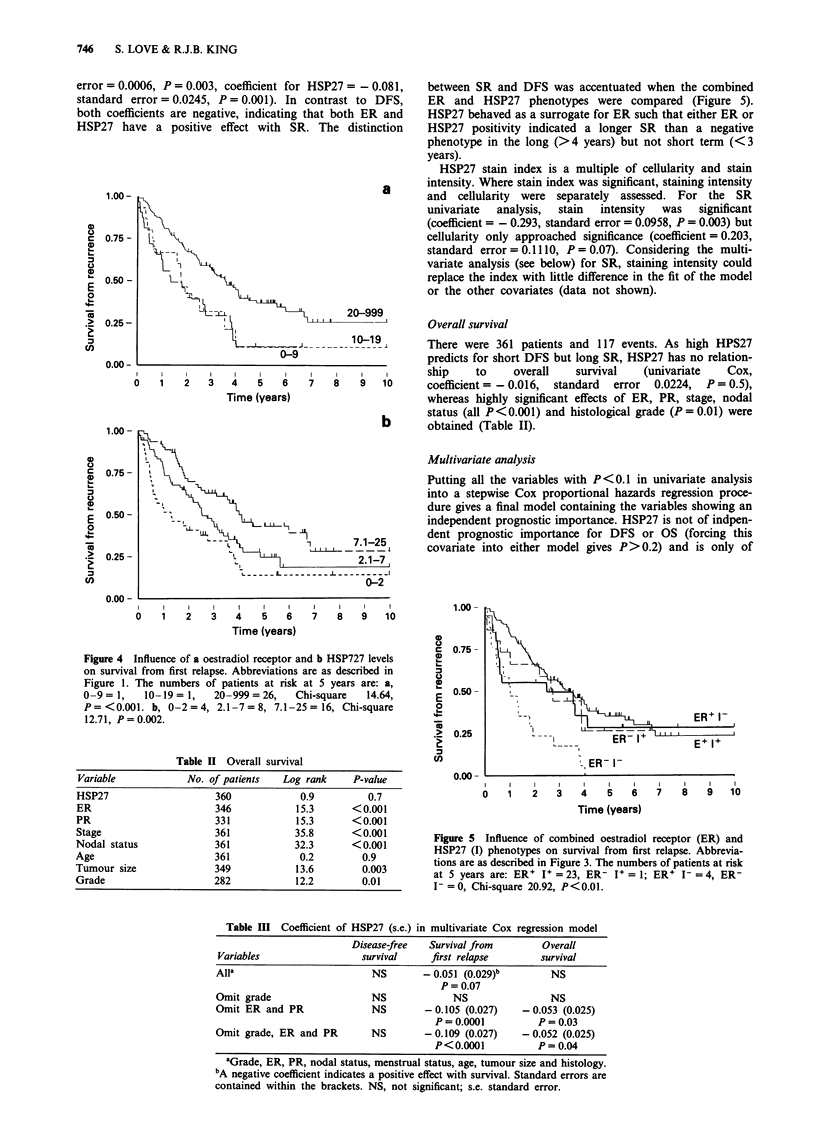

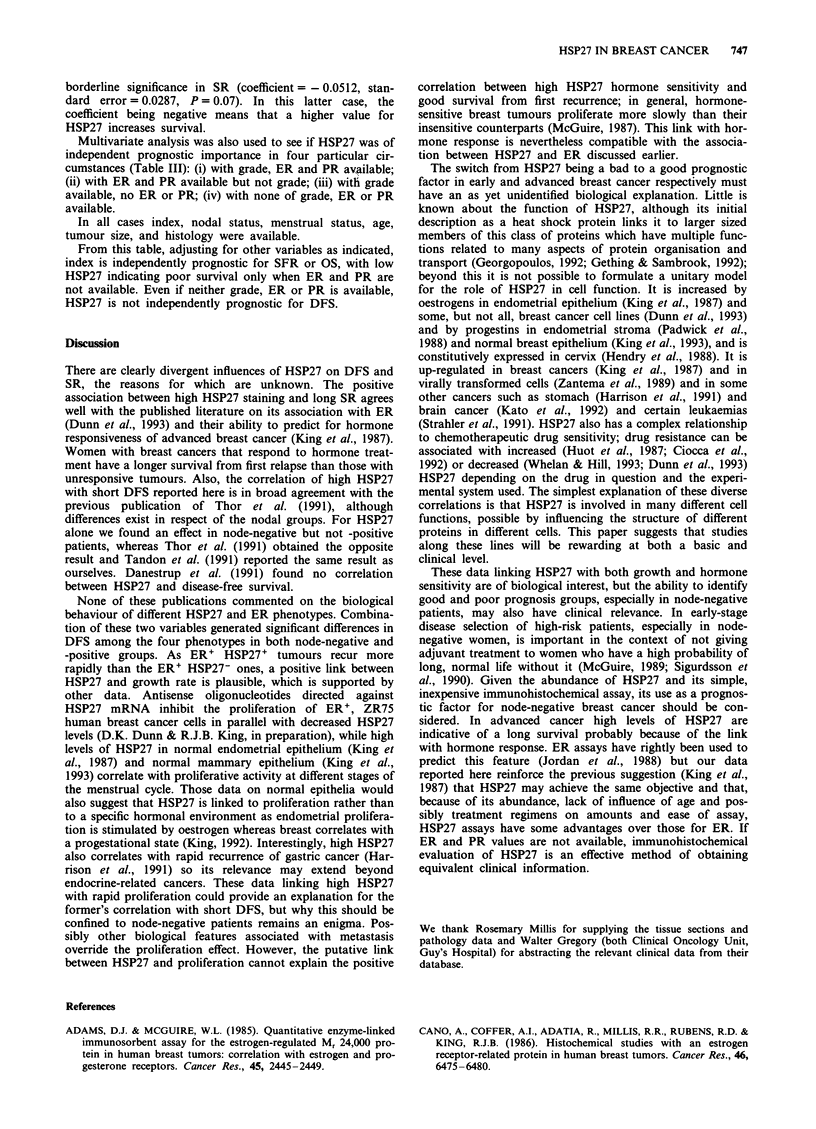

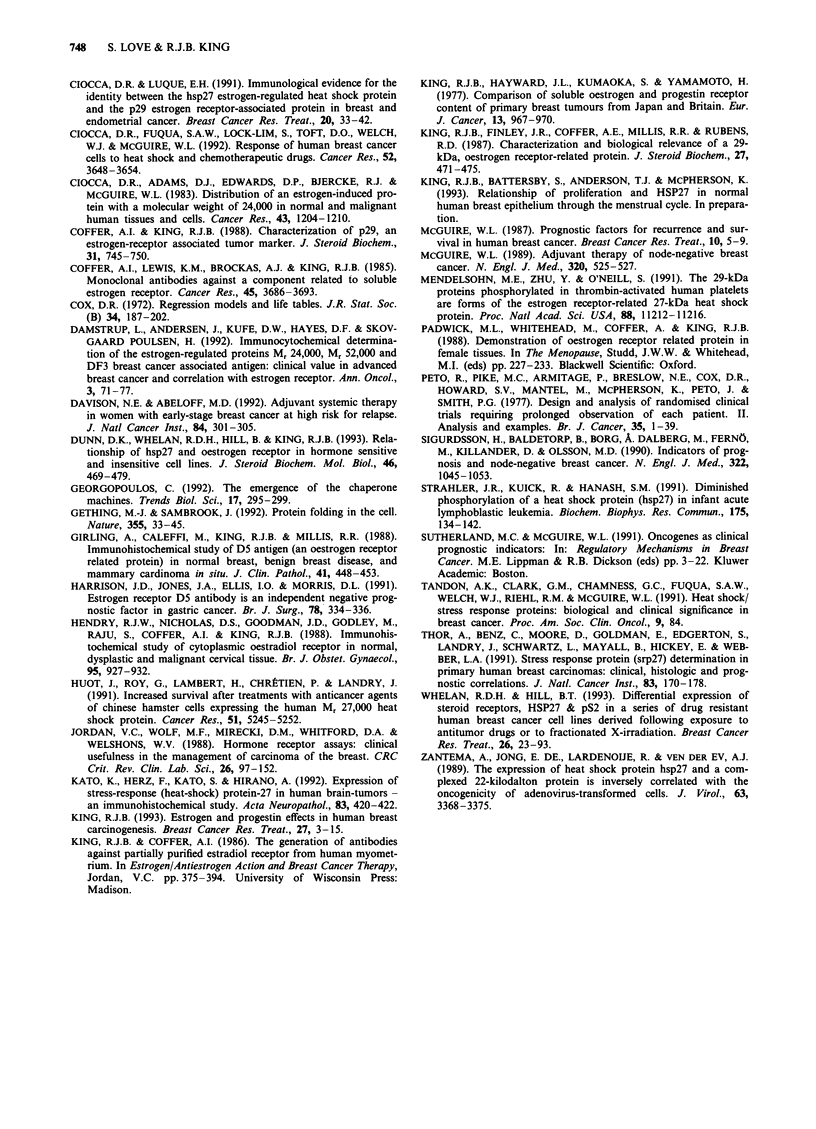


## References

[OCR_00901] Adams D. J., McGuire W. L. (1985). Quantitative enzyme-linked immunosorbent assay for the estrogen-regulated Mr 24,000 protein in human breast tumors: correlation with estrogen and progesterone receptors.. Cancer Res.

[OCR_00907] Cano A., Coffer A. I., Adatia R., Millis R. R., Rubens R. D., King R. J. (1986). Histochemical studies with an estrogen receptor-related protein in human breast tumors.. Cancer Res.

[OCR_00927] Ciocca D. R., Adams D. J., Edwards D. P., Bjercke R. J., McGuire W. L. (1983). Distribution of an estrogen-induced protein with a molecular weight of 24,000 in normal and malignant human tissues and cells.. Cancer Res.

[OCR_00921] Ciocca D. R., Fuqua S. A., Lock-Lim S., Toft D. O., Welch W. J., McGuire W. L. (1992). Response of human breast cancer cells to heat shock and chemotherapeutic drugs.. Cancer Res.

[OCR_00915] Ciocca D. R., Luque E. H. (1991). Immunological evidence for the identity between the hsp27 estrogen-regulated heat shock protein and the p29 estrogen receptor-associated protein in breast and endometrial cancer.. Breast Cancer Res Treat.

[OCR_00933] Coffer A. I., King R. J. (1988). Characterization of p29, an estrogen-receptor associated tumor marker.. J Steroid Biochem.

[OCR_00938] Coffer A. I., Lewis K. M., Brockas A. J., King R. J. (1985). Monoclonal antibodies against a component related to soluble estrogen receptor.. Cancer Res.

[OCR_00947] Damstrup L., Andersen J., Kufe D. W., Hayes D. F., Poulsen H. S. (1992). Immunocytochemical determination of the estrogen-regulated proteins Mr 24,000, Mr 52,000 and DF3 breast cancer associated antigen: clinical value in advanced breast cancer and correlation with estrogen receptor.. Ann Oncol.

[OCR_00955] Davidson N. E., Abeloff M. D. (1992). Adjuvant systemic therapy in women with early-stage breast cancer at high risk for relapse.. J Natl Cancer Inst.

[OCR_00960] Dunn D. K., Whelan R. D., Hill B., King R. J. (1993). Relationship of HSP27 and oestrogen receptor in hormone sensitive and insensitive cell lines.. J Steroid Biochem Mol Biol.

[OCR_00966] Georgopoulos C. (1992). The emergence of the chaperone machines.. Trends Biochem Sci.

[OCR_00970] Gething M. J., Sambrook J. (1992). Protein folding in the cell.. Nature.

[OCR_00974] Girling A., Caleffi M., King R. J., Millis R. R. (1988). Immunohistochemical study of D5 antigen (an oestrogen receptor related protein) in normal breast, benign breast disease, and mammary carcinoma in situ.. J Clin Pathol.

[OCR_00980] Harrison J. D., Jones J. A., Ellis I. O., Morris D. L. (1991). Oestrogen receptor D5 antibody is an independent negative prognostic factor in gastric cancer.. Br J Surg.

[OCR_00985] Henry R. J., Goodman J. D., Godley M., Raju K. S., Coffer A. I., King R. J. (1988). Immunohistochemical study of cytoplasmic oestradiol receptor in normal, dysplastic and malignant cervical tissue.. Br J Obstet Gynaecol.

[OCR_00992] Huot J., Roy G., Lambert H., Chrétien P., Landry J. (1991). Increased survival after treatments with anticancer agents of Chinese hamster cells expressing the human Mr 27,000 heat shock protein.. Cancer Res.

[OCR_00998] Jordan V. C., Wolf M. F., Mirecki D. M., Whitford D. A., Welshons W. V. (1988). Hormone receptor assays: clinical usefulness in the management of carcinoma of the breast.. Crit Rev Clin Lab Sci.

[OCR_01004] Kato M., Herz F., Kato S., Hirano A. (1992). Expression of stress-response (heat-shock) protein 27 in human brain tumors: an immunohistochemical study.. Acta Neuropathol.

[OCR_01025] King R. J., Finley J. R., Coffer A. I., Millis R. R., Rubens R. D. (1987). Characterization and biological relevance of a 29-kDa, oestrogen receptor-related protein.. J Steroid Biochem.

[OCR_01019] King R. J., Hayward J. L., Kumaoka S., Yamamoto H. (1977). Comparison of soluble oestrogen and progestin receptor content of primary breast tumours from Japan and Britain.. Eur J Cancer.

[OCR_01008] King R. J. (1993). William L. McGuire Memorial Symposium. Estrogen and progestin effects in human breast carcinogenesis.. Breast Cancer Res Treat.

[OCR_01040] McGuire W. L. (1989). Adjuvant therapy of node-negative breast cancer.. N Engl J Med.

[OCR_01037] McGuire W. L. (1987). Prognostic factors for recurrence and survival in human breast cancer.. Breast Cancer Res Treat.

[OCR_01044] Mendelsohn M. E., Zhu Y., O'Neill S. (1991). The 29-kDa proteins phosphorylated in thrombin-activated human platelets are forms of the estrogen receptor-related 27-kDa heat shock protein.. Proc Natl Acad Sci U S A.

[OCR_01056] Peto R., Pike M. C., Armitage P., Breslow N. E., Cox D. R., Howard S. V., Mantel N., McPherson K., Peto J., Smith P. G. (1977). Design and analysis of randomized clinical trials requiring prolonged observation of each patient. II. analysis and examples.. Br J Cancer.

[OCR_01063] Sigurdsson H., Baldetorp B., Borg A., Dalberg M., Fernö M., Killander D., Olsson H. (1990). Indicators of prognosis in node-negative breast cancer.. N Engl J Med.

[OCR_01069] Strahler J. R., Kuick R., Hanash S. M. (1991). Diminished phosphorylation of a heat shock protein (HSP 27) in infant acute lymphoblastic leukemia.. Biochem Biophys Res Commun.

[OCR_01075] Sunderland M. C., McGuire W. L. (1991). Oncogenes as clinical prognostic indicators.. Cancer Treat Res.

[OCR_01090] Thor A., Benz C., Moore D., Goldman E., Edgerton S., Landry J., Schwartz L., Mayall B., Hickey E., Weber L. A. (1991). Stress response protein (srp-27) determination in primary human breast carcinomas: clinical, histologic, and prognostic correlations.. J Natl Cancer Inst.

[OCR_01094] Whelan R. D., Hill B. T. (1993). Differential expression of steroid receptors, hsp27, and pS2 in a series of drug resistant human breast tumor cell lines derived following exposure to antitumor drugs or to fractionated X-irradiation.. Breast Cancer Res Treat.

[OCR_01101] Zantema A., de Jong E., Lardenoije R., van der Eb A. J. (1989). The expression of heat shock protein hsp27 and a complexed 22-kilodalton protein is inversely correlated with oncogenicity of adenovirus-transformed cells.. J Virol.

